# Characterization of Outer Membrane Vesicles from *Brucella melitensis* and Protection Induced in Mice

**DOI:** 10.1155/2012/352493

**Published:** 2011-12-29

**Authors:** Eric Daniel Avila-Calderón, Ahidé Lopez-Merino, Neeta Jain, Humberto Peralta, Edgar Oliver López-Villegas, Nammalwar Sriranganathan, Stephen M. Boyle, Sharon Witonsky, Araceli Contreras-Rodríguez

**Affiliations:** ^1^Departamento de Microbiología, Escuela Nacional de Ciencias Biológicas, Instituto Politécnico Nacional, Prolongación de Carpio y Plan de Ayala S/N, Colonia Santo Tomás, 11340, Mexico, DF, Mexico; ^2^Center for Molecular Medicine and Infectious Diseases, Virginia-Maryland Regional College of Veterinary Medicine, Virginia Tech, Blacksburg, VA 24060, USA; ^3^Programa de Genómica Funcional de Procariotes, Centro de Ciencias Genómicas, Universidad Nacional Autónoma de México, Avenue Universidad s/n, P.O. Box 565-A, 62210 Cuernavaca, MOR, Mexico

## Abstract

The outer membrane vesicles (OMVs) from smooth *B. melitensis* 16 M and a derived rough mutant, VTRM1 strain, were purified and characterized with respect to protein content and induction of immune responses in mice. Proteomic analysis showed 29 proteins present in OMVs from *B. melitensis* 16 M; some of them are well-known *Brucella* immunogens such as SOD, GroES, Omp31, Omp25, Omp19, bp26, and Omp16. OMVs from a rough VTRM1 induced significantly higher expression of IL-12, TNF*α*, and IFN*γ* genes in bone marrow dendritic cells than OMVs from smooth strain 16 M. Relative to saline control group, mice immunized intramuscularly with rough and smooth OMVs were protected from challenge with virulent strain *B. melitensis* 16 M just as well as the group immunized with live strain *B. melitensis* Rev1 (*P* < 0.005). Additionally, the levels of serum IgG2a increased in mice vaccinated with OMVs from rough strain VTRM1 consistent with the induction of cell-mediated immunity.

## 1. Introduction

The release of outer membrane vesicles (OMVs) from bacteria is a phenomenon described about 40 years ago. OMVs are released spontaneously during the normal growth of Gram-negative bacteria [1–3]. OMVs have been described in both pathogenic and nonpathogenic Gram-negative bacteria such as *Escherichia coli *[4, 5], *Shigella *spp. [6, 7], *Neisseria *spp. [8], *Porphyromonas* spp. [9], *Pseudomonas aeruginosa *[7], *Helicobacter pylori *[10], *Vibrio *spp. [11], *Salmonella *spp. [12], *Brucella* spp. [13, 14], *Actinobacillus *spp. [15, 16], *Xenorhabdus nematophilus *[17], and *Pseudoalteromonas antarctica *[18].

OMVs possess a bilayer membrane and contain components such as lipoproteins, outer membrane proteins (OMP), lipopolysaccharide (LPS), and some periplasmic components [1–3]. OMVs have been implicated in many processes including the release of virulence factors such as proteases and toxins, signaling between bacterial and eukaryotic cells, DNA transfer, antibacterial activity, immunomodulation of the host, and facilitation of bacterial survival during envelope stress [2, 3, 19, 20].

Other studies have revealed that OMVs trigger the innate inflammatory response. For example, OMVs from *Salmonella typhimurium* are able to activate dendritic cells to secrete IL-12 and TNF*α* [12], and OMVs from *Pseudomonas aeruginosa* and *Helicobacter pylori* are able to elicit IL-8 production by epithelial cells [21, 22].

The use of OMVs from different Gram-negative bacteria as acellular vaccines has been explored in recent years [23–26]. OMV vaccines have been effective in the specific case of serogroup B of *Neisseria meningitis* [24]. More recently, OMVs from *Vibrio cholerae* and *Bordetella pertusis *were demonstrated to elicit protection in mouse model [26, 27]. The interest in OMVs as vaccine carriers is increasing, and recent reports have showed that engineered OMVs were able to harbor overexpress antigens [28].

Brucellosis is a worldwide spread zoonotic disease transmitted from domestic animals to humans. It is frequently acquired by ingestion, inhalation, or direct contact of conjunctiva or skin-lesions with infected animal products. The human disease represents an important cause of morbidity worldwide whereas animal brucellosis is associated with serious economical losses caused mainly by abortion and infertility in ruminants [29].

The first effective *Brucella* vaccine was based on live *Brucella abortus* strain 19 (S19), a smooth strain attenuated by an unknown process induced by its subculturing. This strain induces reasonable protection against *B. abortus* in cattle, but at the expense of persistent serological responses that confound differential serodiagnosis of vaccinated and field- infected cattle. A similar problem occurs with the *B. melitensis* Rev.1 strain that is still the most effective vaccine against caprine and ovine brucellosis. This problem has been overcome in cattle by the development of the rifampicin-resistant mutant *B. abortus* RB51 strain. This strain has been proven safe and effective in the field against bovine brucellosis and exhibits negligible interference with diagnostic serology [30].

Currently, smooth live attenuated vaccines *B. abortus *S19 and *B. melitensis *Rev1 as well rough live attenuated vaccine *B. abortus *RB51 are used in the control of animal brucellosis. These smooth vaccines for animals may cause disease and considered unsuitable for use in humans; the rough strain RB51 is rifampin resistant and is considered unsuitable for humans as rifampin is one of the antibiotics of choice for therapy [31]. In the last few decades much research has been done in the attempt to develop safer *Brucella *vaccines [32]. It is important to mention that there is no licensed vaccine for prevention of human brucellosis. A human vaccine could be useful to protect farmers, veterinarians, animal care workers, and general populations living in endemic brucellosis areas [30].

Since OMVs from other bacteria have been used for development of acellular vaccines, we were interested in assessing the protective immune response induced by *Brucella* OMVs. The first studies related to OMVs isolated from *Brucella* spp. were limited to analysis of their protein profile using one-dimensional SDS-PAGE [14, 33]. More recently, Omp25 and Omp31 were identified in *B. suis *OMVs using monoclonal antibodies [13]. In 2007, Lamontagne et al. performed a proteomic analysis of a fraction they called outer membrane fragments from virulent *B. abortus* 2308 and attenuated BvrR/BvrS mutants [34]. To date the composition of OMVs from *B. melitensis* has not been yet explored.

In the attempt to increase the current understanding of the composition of *B. melitensis* OMVs, the proteome of OMVs isolated from smooth* B. melitensis *16 M is described. Because of the distinct immunological role of the *Brucella *O-side chain in the host, mice were immunized with OMVs purified from smooth *B. melitensis* 16 M and the rough mutant *B. melitensis* VTRM1 (lacking the side O chain of LPS). The difference in dendritic cell cytokine expression and the serum IgG subtypes levels as well as the level of protection afforded to mice is also described.

## 2. Materials and Methods

### 2.1. Ethics Statement

The mice experiments were approved and conducted by Institutional Animal Care and Use Committee (approved protocol and 07-047CVM) at Virginia Tech.

### 2.2. Bacterial Strains and Growth Conditions

*B. melitensis *16 M (ATCC 23456) and *B. melitensis* VTRM1 rough mutant derived from* B. melitensis *16 M [35] were used. Both strains were cultured on tryptic soy agar (TSA) plates supplemented with 0.7% yeast extract and incubated 36 h at 37°C. A bacterial suspension was obtained from both strains, adjusted each to 0.5 g of cells per mL of tryptic soy broth, of which 0.5 mL was spread onto each of 100 TSA plates (10 cm diameter) and incubated at 37°C for 48 h.

### 2.3. OMVs Purification

The OMVs purification was performed according to the protocol described by Gamazo et al., 1989. Briefly, the bacteria were harvested with a rubber policeman and suspended in 250 mL sterile phosphate-buffered saline (PBS 0.1 M, pH 7.3). The bacterial suspension was centrifuged at 10,000 ×g for 30 min. The supernatant was passed through a 0.22 *μ*m filter (Millipore Corp.), and a sterility test was performed by culturing an aliquot onto a TSA plate followed by incubation for 72 h at 37°C; the filtrate was stored at 4°C during the viability check. The filtered supernatant was centrifuged at 100,000 ×g for 2 h at 4°C. The pellet was washed twice with 25 mL of sterile PBS, and the OMVs were resuspended in 1 mL of sterile PBS. The total protein concentration was determined using PIERCE-BCA (PIERCE) reagents as per manufacturer's recommendations. The OMVs samples were divided into 0.5 mL aliquots and stored at −20°C until used [33].

### 2.4. Bone Marrow-Derived Dendritic Cells (BMDC)

Dendritic cells were derived from 8 wk old, female BALB/c mice by *in vitro* culture of bone-marrow cells with 20 ng/mL rGM-CSF for 7 days as previously described [36, 37]. On day 7, cells showed differentiated morphology (BMDC) and expressed DC markers (CD11c+) in 75% of the population as assessed by flow cytometry (data not shown).

### 2.5. In Vitro Stimulation of BMDC, RNA Extraction, and Reverse-Transcription Polymerase Chain Reaction

Aliquots of 2.5 × 10^6^ BMDC per well were plated in a 6-well flat-bottomed plate by triplicate and incubated overnight. Then 40 *μ*g of purified OMVs from smooth *B. melitensis* 16 M or OMVs from rough *B. melitensis* VTRM1 were added to each well by triplicate. Total RNA (RNAeasy Qiagen) was extracted from BMDC (stimulated and unstimulated) at 1, 3, 6, and 12 h after induction. The DNA was removed with DNase I (DNA-free Kit Ambion). Then cDNA was prepared from 1 *μ*g of total RNA (Promega, A2500 kit). To verify the complete elimination of DNA, PCR for glyceraldehyde-3-phosphate dehydrogenase (*GAPDH*) gene amplification was performed (data not shown).

### 2.6. Real Time-PCR

Templates cDNA were analyzed for IL-2, IL-6, IL-12p40 (IL-12), IL-10, IL-17, IL-23, INF-*γ*, TNF-*α*, and TGF-*β* (SABioscienes) expression using the PCR Array and the RT2 SYBR Green/Fluorescein qPCR Master Mix (SABiosciences) on the iCycler PCR System (Bio-Rad) as per recommendations of the manufacturer. Fold changes in gene expression were calculated using the ΔΔCt method in the PCR Array Data Analysis template. The amplification of house-keeping *gapdh* gene was used to normalize the fold changes in the cytokine expression.

### 2.7. Mice Immunizations

Female BALB/c mice of 6 weeks of age (5 per group) were vaccinated by two intramuscular inoculations, at day 0 and day 30, with 5 *μ*g of purified OMVs from *B. melitensis* 16 M and *B. melitensis* VTRM1. Before the first dose, mice were prebled by puncturing retro-orbital plexus under anesthesia. Two weeks after boosting, the mice were bled by the same route. The serum was separated from the clotted blood and stored at −20°C until use for detection of IgG subtypes. As a positive control, a group of mice was vaccinated with 1.5 × 10^4^ CFU of vaccine strain, *B. melitensis* Rev1. As a negative control, one group of mice was injected with saline. Mice were challenged at 6 weeks after the first vaccination dose with 5 × 10^4^ CFUs of virulent strain *B. melitensis* 16 M by intraperitoneal route. At 2 weeks after challenge, all the mice were euthanized by CO_2_ asphyxiation followed by cervical dislocation, spleens were collected aseptically, and colony-forming units (CFU) were determined.

### 2.8. Indirect ELISA

Levels of serum immunoglobulin IgG1 and IgG2a isotypes with specificity to OMVs from smooth *B. melitensis* 16 M and rough strain VTRM1 were determined by indirect ELISA. Sera from mice immunized with OMVs purified from *B. melitensis* 16 M were tested with OMVs purified from *B. melitensis *16 M, and sera from mice immunized with OMVs purified from rough *B. melitensis* VTRM1 were tested with OMVs purified from rough strain. The OMVs were diluted in carbonate buffer, pH 9.6. The wells of polystyrene plates (Nunc-Immunoplate with maxisorp surface) were coated with 100 *μ*L/well of the diluted antigens (2.5 *μ*g/mL of protein from OMVs). Following overnight incubation at 4°C, plates were washed four times in wash buffer (Tris-buffered saline at pH 7.4,  .05% Tween 20) and blocked with 2% bovine serum albumin (BSA) in Tris-buffered saline. After 1 h incubation at 37°C, mice sera with appropriate dilution in blocking buffer were added to the wells (50 *μ*L/well). Each serum sample was tested in triplicate wells; the plates were incubated for 4 h at room temperature and washed four times. Horse radish peroxidase-labeled anti-mouse isotype-specific conjugates (Southern Biotechnology Associates Inc, Birmingham, Alabama) were added (50 *μ*L/well) at an appropriate dilution. After 1 h incubation at room temperature, the plates were washed four times. A 100 *μ*L of substrate solution (TMB Microwell peroxidase substrate; Kirkegaard and Perry Laboratories, Gaithersburg, Md) was applied to each well. After 20 min incubation at room temperature, the enzyme reaction was stopped by adding 100 *μ*L of stop solution (0.185 M sulfuric acid), and the absorbance at 492 nm was recorded using microplate reader (Molecular Devices, Sunnyvale, Calif).

### 2.9. Electron Microscopy

20 *μ*L of purified OMVs (25 *μ*g of protein) or intact bacteria were placed onto copper grids coated with formvar and dried using filter paper. 40 *μ*L of 1% phosphotungstic acid was added, and the grids were allowed to stand overnight at room temperature; they were observed under the transmission electron microscope (JEOL model JEM 10-10).

### 2.10. Denaturing Gel Electrophoresis

SDS-PAGE was performed in 15% acrylamide slab gels by the method of Laemmli [38]; the gels were stained with Coomassie blue. The apparent molecular masses of the OMV proteins were determined by comparing their electrophoretic mobility with that of the wide-range molecular mass markers [SigmaMarker (Sigma)] using the computer program SigmaGel V. 1.0.

### 2.11. Enzymatic Digestion

After the separation of OMV proteins by denaturing electrophoresis, the acrylamide gel was cut into six sections. The excised samples were reduced with 50 mM dithiothreitol, alkylated with iodoacetamide and then “in gel” digested with trypsin. The peptides were desalted using a ZipTip (Millipore Corp) and then concentrated in a Speed-Vac SPD 1010 ThermoElectron (Instituto Nacional de Biotecnologia-UNAM, Cuernavaca, México).

### 2.12. LC-MS/MS

The samples were reconstituted to approximately 0.1–0.5 *μ*g/*μ*L in 50% acetonitrile containing 1% acetic acid and placed directly into a Finnigan LCQ ion trap mass spectrometer (Instituto Nacional de Biotecnologia-UNAM, Cuernavaca, Mexico), using a Surveyor MS syringe pump delivery system. The eluate at 10 *μ*L/min was split to allow only 5% of the sample to enter the nanospray source (0.5 *μ*L/min). LC-MS/MS analyses were carried out using a PicoFrit needle/column RP C18 from New Objective (Woburn, Mass, USA), with a fast gradient system from 5% to 60% of solution B (100% acetonitrile containing 1% acetic acid) for a period of 45 min.

The electrospray ionization source voltage was set at 1.8 kV and the capillary temperature at 130°C. Collision-Induced Dissociation (CID) was performed using 25 V of collision energy, 35–45% (arbitrary units) of normalized collision energy and the scan had the wide band activated.

All spectra were obtained in the positive-ion mode. Data acquisition and the deconvolution of data were carried out using Xcalibur software on a Windows XP PC system. The MS/MS spectra from enzymatically generated peptides were analyzed by Sequest software from Finnigan (Palo Alto, Calif) and MASCOT search engine from Matrix Science Ltd (Boston, Mass).

### 2.13. In Silico Analysis

Once the proteins in the OMVs were identified, an *in silico* analysis was performed. Initially the amino acid sequences of the identified proteins were analyzed by BLAST comparing them to similar sequences from species of *Brucella* and other bacteria (http://www.ncbi.nlm.nih.gov/BLAST/). The isoelectric point and molecular weight were determined using Antheprot 2000 V. 5.2.

The prediction of “motif” sequences was performed by searching My Hits Motif Scan database (http://hits.isb-sib.ch/cgi-bin/PFSCAN) that uses different databases such as PROSITE, HAMAP, and Pfam. The prediction of the subcellular location of the proteins was carried out on the PSORT server available at http://www.psort.org [39] and Softberry database. The MatGAT V. 2.02 [40] program was used to determinate the similitude grade of the homologous sequences found by the BLAST search. The ProLinks database (http://dip.doe-mbi.ucla.edu/pronav/) [41] and Gene Ontology (http://www.geneontology.org) [42] were used to determinate the hypothetical function of proteins into OMVs.

### 2.14. Statistical Analysis

Statistical analysis, Two-Way-ANOVA, was carried out with SigmaStat statistical package V. 2.0 (SYSTAT).

## 3. Results

### 3.1. Isolation of OMVs and Electron Microscopy

**Figure 1:**
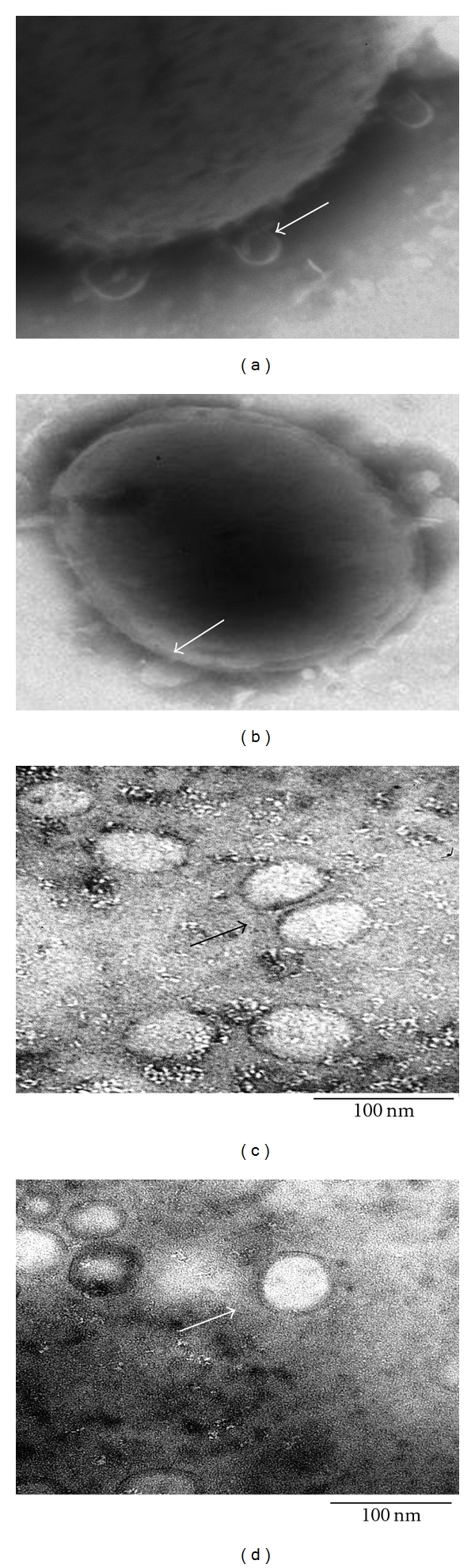
Electron microscopy of OMVs from smooth *B. melitensis* M16 and rough *B. melitensis *VTRM1. Negative stain of OMVs released from surface *B. melitensis *VTRM1 (a) and *B. melitensis* 16 M (b); negative stain of OMV purified by differential centrifugation from both strains; *B. melitensis *VTRM1 (c) and *B. melitensis* 16 M (d). The arrows point to the apparent shedding of the OMVs from the cell surface in (a) and (b); while the arrows in (c) and (d) point to spherical OMVs purified from both strains. The bars correspond to 100 nm.

OMVs were isolated from cell-free culture medium by differential centrifugation. In order to confirm purification of the OMVs from both strains, electron micrographs were performed using negative staining with phosphotungstic acid. In the micrographs (Figure 1), it is possible to see the bleb formation leading to the liberation of OMVs from the outer surface of the *Brucella*. In addition, the spherical morphology (average diameter 60–90 nm) of the purified OMVs including a double membrane can be observed as previously described [14, 33]. Moreover, no membrane debris bigger than 100 nm were observed by electron microscopy. In general, no differences, at least in the shape or in the number of OMVs released, were observed for the smooth or rough *Brucella* strains.

### 3.2. Cytokine Expression

**Figure 2:**
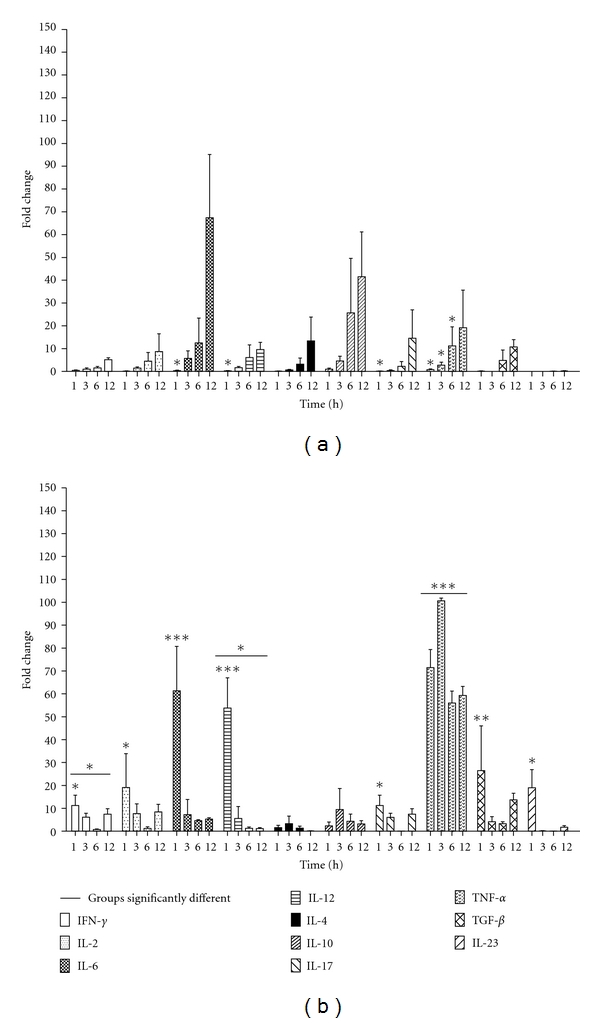
Cytokine expression of BMDC stimulated with OMVs. (a) BMDC stimulated with OMVs from smooth *B. melitensis* 16 M. (b) BMDC stimulated with OMVs from rough *B. melitensis *VTRM1. Two-way ANOVA analysis was performed to compare results. *(*P* < 0.05); **(*P* < 0.01); ***(*P* < 0.001).

In order to explore if OMVs could induce an immune response in antigen-presenting cells, we used BMDC exposed to OMVs either from smooth or rough *B. melitensis*. At increasing times following exposure, the cytokines associated with the DC1-mediated Th1 (IFN-*γ*, IL-2, IL-6, IL-12, and TNF-*α*), DC2-mediated Th2 (IL-4 and IL-10), and DC17-mediated Th17 (IL-17, IL-23, and TGF-*β*) responses were measured by qRT-PCR. The results are shown in Table S1 (see in table S1 Supplementary Material available online at doi: 10.1155/2012/352493). The cytokine profiles elicited in the BMDC stimulated with smooth *B. melitensis* 16 M OMVs reached a maximum at 12 hours following stimulation with the highest production of IL-6, IL-4, IL-10, and IL-17 (Figure 2(a)). In contrast, the induction of cytokines by rough OMVs reached maximum expression at 1 h after stimulation and decreased over time, except for IL-10 (maximum expression at 3 hours) and for TNF-*α* (maximum expression at 3 hours) (Figure 2(b)). Using statistical analysis we compared the cytokine expression obtained from BMDC stimulated with OMVs from smooth and rough *Brucella* strains (Two-way-ANOVA analysis). Results showed significant differences between the cytokine profile induced by OMVs purified from rough and smooth *B. melitensis *(*P* < 0.05). OMVs from rough *B. melitensis* VTRM1 induced three cytokines that were significantly higher: IFN-*γ*, TNF-*α*, and IL-12 (*P* < 0.05, *P* < 0.01 and *P* < 0.001, resp.).

### 3.3. Protection against Challenge with Virulent *B. melitensis* 16 M

**Figure 3:**
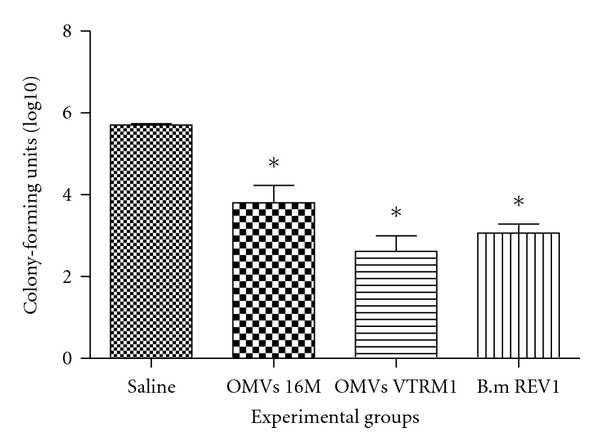
Analysis of IgG1 (a) and IgG2a (b) antibody responses of BALB/c mice to outer membrane vesicles from *Brucella*. Outer membrane vesicles (OMVs) were purified from *B. melitensis* 16 M and *B. melitensis *VTRM1, and mice were immunized. Sera from each mouse were collected and were assayed individually by ELISA. Antibody levels are expressed as optical density (OD) at 492 nm.

Mice were challenged with virulent strain *B. melitensis* 16 M to examine the protection induced by OMVs. In this experiment, protection was defined as a significant reduction in the number of bacteria in the spleens of immunized mice compared to the mice receiving saline. The *B. melitensis* Rev1 vaccine induced 2.64 log units of protection compared to saline control (Figure 3). In the case of mice vaccinated with OMVs, we observed that smooth OMVs induced 1.9 log units, and rough OMVs induced 3.08 log units of protection compared to saline control (*P* < 0.005).

### 3.4. Serology

**Figure 4:**
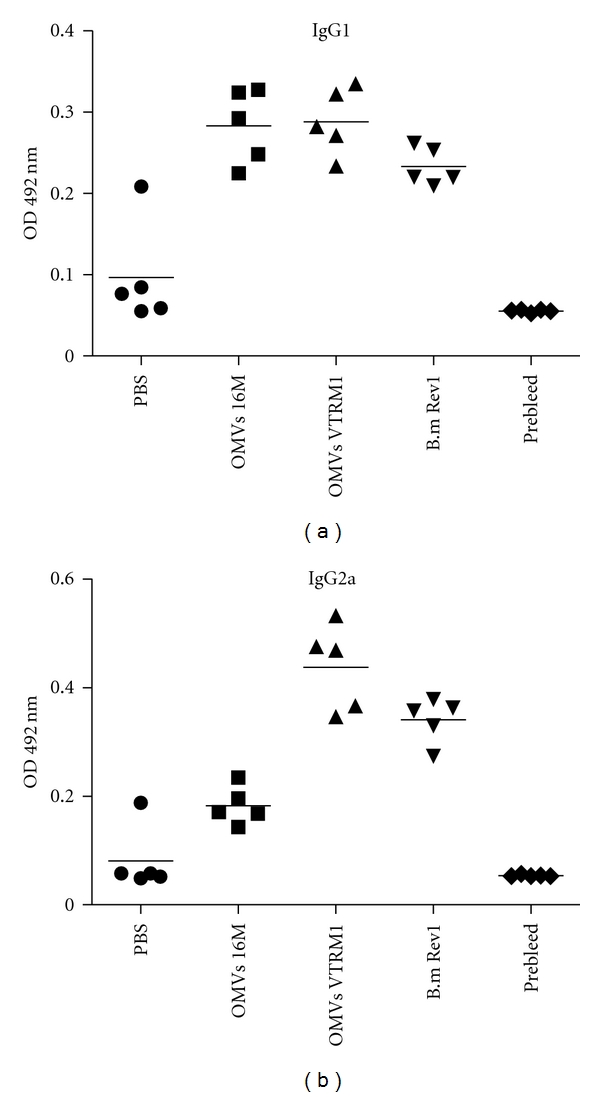
Level of protection against *B. melitensis *16 M conferred by outer membrane vesicles purified from *B. melitensis* 16 M and *B. melitensis* VTRM1. In this experiment vaccine strain *B. melitensis* Rev1 was used as a positive control of vaccination, as negative control was used saline. *n* = 5 (*P ≤ 0.005 comparisons were OMVs versus saline, and *B. melitensis *Rev1 versus saline).

Presence of antibodies specific to OMVs in serum of the mice vaccinated was determined by ELISA. The induction of IgG2a subclass during immune response should give an idea about Th1 or Th2 balance. As illustrated in Figures 4(a) and 4(b) OMVs purified from rough *B. melitensis* VTRM1 induced a higher IgG2a response than IgG1, suggesting a shift toward a Th1 response. In contrast, OMVs from smooth strain *B. melitensis* 16 M induced the same levels of IgG1 and IgG2a.

### 3.5. SDS-PAGE and Proteomic Analysis

**Figure 5:**
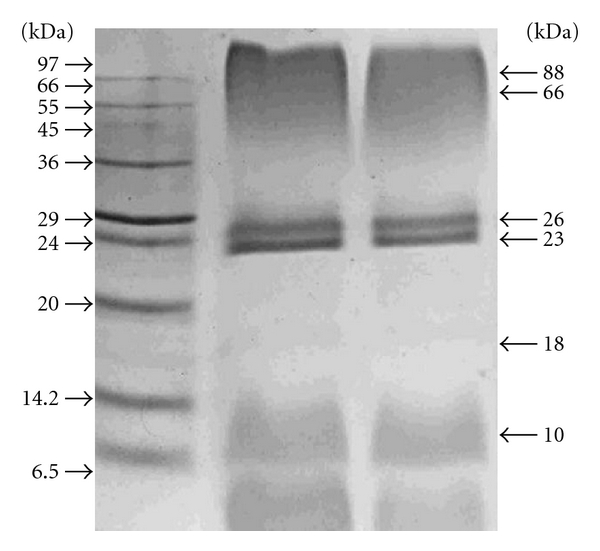
Electrophoretic profiles of OMVs purified from *B. melitensis*. Lane 1, molecular weight markers; lane 2, OMVs from smooth *B. melitensis *16 M (80 *μ*g); lane 3, OMVs from rough *B. melitensis *VTRM1 (80 *μ*g). SDS-PAGE gels were stained with Coomassie blue.

The denatured electrophoretic protein profiles observed from OMVs obtained from rough *B. melitensis* VTRM1 and smooth *B. melitensis* 16 M show no discernable differences (Figure 5). The trypsin-generated peptide masses, as well as their fragment ions, were analyzed by LC-MS/MS. The resulting peptides sequences were used to query databases that led to the identification of 29 unique proteins (Table 1). A query result was only considered as significant if the overall score was higher than 25 and more than two tryptic peptides as well as their fragment ions matched to the protein and the calculated molecular weight corresponded to molecular weight in the original gel section [43].

### 3.6. In Silico Analysis

The results of the proteomic characterization of the *B. melitensis* vesicular proteins are summarized in Table 1. In addition, the complete results are available in supporting information Table S2 and Table S3. The identification score varied from 55 to 100%, with coverage from 3 to 50%. Besides the identification of each spot and calculation of its basic biochemical characteristics, the isoelectric point and molecular weight was obtained by means of analysis and search in databases. Subcellular location, protein motifs, immunogenic regions, signal peptide prediction, and closest homologues were also analyzed. Of the 29 proteins identified, approximately 52% belonged to the outer membrane, 17% to the periplasm, 20.6% to the cytoplasm, 2 proteins were from inner membrane, and 1 protein was predicted to be an extracellular protein. Using the Motif databases these proteins were classified into five groups: (i) structural and transport proteins (such as the outer membrane proteins), (ii) antigenic proteins, (iii) involved in metabolic processes (e.g., Frr, HU, GroES), (iv) involved in stress response (e.g., Dps, TrxC, and SOD), and v) invasion proteins (e.g., InvB and IalB). About 60% of the proteins were predicted to contain signal peptides and thus capable to be exported or targeted by the cellular machinery to the periplasm or outer membrane. Only twelve proteins did not contain signal peptides, five of these were predicted as cytoplasmic or mature periplasmic proteins in which the signal sequence was processed.

Additionally, the analysis of sequences using ProLinks [41] showed that with the exception of two proteins, the possible functions of 27 proteins were predicted. While the analysis of sequences using Gene Ontology terms showed that one half (14/29) of the proteins appeared to be involved in transport and/or integrity of the membrane.

All the proteins found in the *B. melitensis* OMVs were highly related to homologous proteins in other *Brucella* species (from 88 to 100% in similarity, data not shown). For most proteins, the closest non-*Brucella *homologues were found in *Rhizobia*, such as *Rhizobium* and *Bartonella *(50% to 90% in similarity). Interestingly, four proteins with a high degree of homology to those in *Escherichia coli* were found: FrpB (an iron-regulated outer membrane protein), a metal chelate outer membrane receptor, Dps (involved in DNA protection due to starvation or stationary phase), and SOD (a Cu/Zn superoxide dismutase).

## 4. Discussion

As has been described in other Gram-negative bacteria, OMVs are released from the *Brucella*'s outer membrane as we observed by electron microscopy (Figure 1). The OMVs are shed from both rough and smooth *Brucella* strains, grown in liquid or on solid media, and spontaneously released during the growth as observed previously [13, 14, 33].

OMVs can also strongly activate the host innate and acquired immune response pathways [23]. Based on this previous evidence, we stimulated BMDC with OMVs isolated from smooth and rough *Brucella* strains. At different time points, cytokines expression for DC1-mediated Th1 (IFN-*γ*, IL-2, IL-6, IL-12, and TNF-*α*), DC2-mediated Th2 (IL-4 and IL-10), and DC-mediated Th17 (IL-17, IL-23 and TGF-*β*) was analyzed by qRT-PCR. Interestingly, we determined that OMVs from rough *B. melitensis* VTRM1 induced significantly higher expression of IFN-*γ*, TNF-*α*, and IL-12.

TNF-*α* is necessary for full expression of the macrophage anti-*Brucella* activities. It also plays an important role in the triggering of specific immunity against several intracellular pathogens and positively controls early expression of IL-12 and IFN-*γ* in *Brucella*-infected mice [44]. Our results demonstrated an earlier expression of IL-12 and IFN-*γ* by OMVs from rough *B. melitensis* VTRM1 (Figure 2(b)). Clearly TNF-*α* participates in the establishment of acquired immunity of the Th1 response, with the generation of IFN-*γ*-producing CD4^+^ cells and CD8^+^ cytotoxic cells, two outcomes crucial for the complete killing of the intracellular *Brucella*. TNF-*α* produced a synergistic effect in presence of IFN-*γ* for the ultimate clearance of the infection [45].

The early expression of IL-6, IL-23, and TGF-*β* genes was observed in BMDC stimulated with OMVs from rough *B. melitensis* VTRM1. In contrast, in the case of smooth *B. melitensis* 16 M OMVs, the same cytokines genes were expressed after a longer time with a maximum expression at 12 h following induction. The early induction of the cytokine expression could offer a fast immune response against *Brucella*, for example, if IL-6 promotes a proinflammatory environment. However, the role of the IL-23 and TGF-*β* as part of the Th17 response in *Brucella *infection is not very clear to date. Some data suggest that a Th17 response is favored when IL-6 is present in high quantities, especially in aged mice [46].

The determination of whether Th17 response is merely present as epiphenomena or truly playing a role in the host defense is a focus of current research. We speculate that these differences could be the effect of the O-side chain in the LPS present in the OMVs from the smooth strain but not in the OMVs derived from rough *B. melitensis* VTRM1. In contrast to the infection with smooth *B. abortus *and *B. suis *strains and purified smooth LPS, infection of human DCs with rough mutants of *Brucella* leads to both phenotypic and functional maturation of infected cells [47].

Recently, Surendran et al. observed phenotypic maturation and production of IL-12 and TNF-*α* when murine BMDC were stimulated with live *B. abortus* RB51, a rough vaccine strain approved by the USDA for use in cattle and with *B. abortus *RB51SOD, which overexpressed a Cu-Zn superoxide dismutase (SOD) [37]. In contrast no maturation or secretion of either cytokine occurred when *B. abortus* strain 2308 was used to stimulate murine BMDC [47]. While Billard et al., [47] observed higher production of TNF-*α* and human DC maturation using rough mutants but not with smooth strains of *B. suis,* our cytokine expression results are in agreement with the works published by Billard et al., and Surendran et al. Those are in conflict with the reports by Zwerdling et al. and Macedo et al., where it was observed that smooth *B. abortus *exposure induced human and murine DC maturation, respectively [48]. Zwerdling et al. could only speculate the reasons for these discrepancies and some considerations were made such as: the different method for cell isolation, the concentration of the DC and the interactions between themselves in each experiment, and the type of strains used for those experiments [48].

The direct effect of purified rough or smooth LPS molecules on human DC maturation also has been explored [47]. However no difference could be determined between DC responses to rough or smooth LPS of *B. abortus*. These results are in line with the very low endotoxic properties of *Brucella* LPS and with the equivalent stimulation of macrophages by rough and smooth LPS [47]. This means that the ability of rough *Brucella *strains to induce human or murine DC maturation is not related to a direct effect of their LPS but maybe the absence of the O-side chain could allow the exposure of bacterial surface molecules that should normally be hidden. It is also possible that the absence of O-side chain in the rough OMVs permits a higher exposure to the outer membrane proteins. For example, it is well known that Omp16 and Omp19 interact with the TLR2 receptor and induce the production of IL-12, which is important for the control of infection [44].

Recently, it was demonstrated that Omp16 requires TLR4 interaction for the activation of DC and macrophages and elicits a Th1 and protective immune response [49]. On the other hand TLR4 is not involved in the Th1 and protective response induced by Omp19 [32].

After intramuscular administration of OMVs from *B. melitensis* 16 M and rough *B. melitensis* VTRM1, challenge with virulent *B. melitensis* 16 M was performed in BALB/c mice. Relative to saline control group, mice immunized intramuscularly with rough OMVs were protected from challenge with strain virulent *B. melitensis* 16 M just as well as the groups immunized with live strains *B. melitensis *Rev1 or OMVs from rough strain *B. melitensis* VTRM1 (*P* < 0.005) (Figure 3).

In addition to protection, in this study, we analyzed the humoral immune response induced in mice by OMVs isolated from smooth and rough *Brucella *strains. Since the subclass of IgG response is determined by the pattern of cytokines secreted by CD4 helper T cells, we measured the titers of both the IgG1 and IgG2a antibodies produced against OMVs. As has been observed (Figure 4(b)), OMVs from the rough strain were able to induce a higher IgG2a subclass. This IgG2a isotype is important because of the binding of their Fc portion to Fc receptors on the surface of phagocytes that activates a broad spectrum of antimicrobial responses (e.g., phagocytosis, cytokine synthesis, release of inflammatory mediators, and generation of reactive oxidant species) [50].

The information related to the immune response of OMVs from *Brucella* is limited to observe the antigenicity of these entities in rabbits as reported previously [33]; on the other hand using monoclonal antibodies other authors were able to identify two proteins in OMVs from *B. suis* [13]. In the same line, there are no reports regarding the immune protection of vaccines based on OMVs against *Brucella* infection in mouse model.

Many vaccine candidates for human brucellosis involve live attenuated *Brucella* strains, subunits vaccines, recombinant proteins, and DNA vaccines which have shown to be protective in a mouse model [31].

The biophysical properties of vesicles, as heterogeneous, proteinaceous, amphipathic structures, may allow greater movement through tissues. As a result, vesicles could travel deeper into tissues where resident phagocytes are located [2]. As mentioned previously, the practical application of OMVs as acellular vaccines has been exploited in other pathogens [24, 26, 27]. The advantage to make vaccines based on *Brucella* OMVs could be that they are able to carry many antigens exposed naturally in the outer membrane and periplasm of *Brucella*; on the other hand, OMVs are acellular entities that could give an alternative to make safer vaccines instead of using live *Brucella* vaccines, which have the additional potential complications of replication, virulence, and side effects due to induction of a strong immune response (i.e., fever).

Proteomics approaches have been used to identify the protein components of vesicles in attempts to provide clues to the mechanisms of vesicles production and cargo loading [51]. In this study conventional SDS-PAGE coupled to LC-MS/MS was used to identify the composition of *B. melitensis* 16 M OMVs. The denatured electrophoretic protein profiles observed from OMVs obtained from rough *B. melitensis* VTRM1 and smooth *B. melitensis* 16 M showed no discernable differences (Figure 5). The method selected for OMVs purification was that one reported for *Brucella* spp by Gamazo et al. in 1989, in which the authors observed a range of sizes in OMVs when *Brucella* was grown in solid media [33]. We also observed a slightly better yield of OMVs when solid medium was used instead of liquid medium to grow* Brucella*.

In comparing our results with Gamazo and Moriyon [33], in 1987, they observed an electrophoretic profile that showed two major bands (25 and 30 kDa) and several minor bands (18, 22, and 84 kDa) in the OMVs of both smooth *B. melitensis* 16 M and a rough strain *B. melitensis* B115. Our results showed bands similar to those observed by Gamazo and Moriyon, [14] in both smooth and rough OMVs. However, the same research group in 1989 working with OMVs purified from field strains of *B. melitensis* and *B. ovis* observed different electrophoretic profiles divided in four groups of proteins: A (25.5–32 kDa), B (21.5–22.5 kDa), C (18–19.5 kDa), and D (13–15.5 kDa); these profiles were different from that reported in 1987. The differences may be due to inherent differences between field strains compared with the reference strain.

After electrophoretic separation, the OMVs proteins from *B. melitensis* 16 M were excised and digested with trypsin. The trypsin-generated peptide masses, as well as their fragment ions, were analyzed by LC-MS/MS. The resulting peptides sequences were used to query databases led to the identification of 29 unique proteins (Table 1). Our results showed that the outer membrane proteins are the principal components of *Brucella* OMVs as has been reported previously for other Gram-negative bacteria [23, 41, 52]. However, Lamontagne et al. (2007) found that periplasmic and cytoplasmic proteins as the principal components of OMVs from *B. abortus *while outer membrane proteins were present as a smaller proportion.

In OMVs from *B. melitensis* 16 M we identified Omp25 and Omp31 that belong to the major outer membrane protein family [53]. Boigegrain et al. (2004) identified Omp25 and Omp31 in OMVs from *B. suis* using monoclonal antibodies. Also, Lamontagne et al. (2007) using mass spectrometry were able to identify the Omp31b and Omp25 (Omp3a) in OMVs from *B. abortus*. The spontaneous release of the OMVs and gentle isolation procedures should minimize cytoplasmic leakage and prevent the contamination that follows cell disruption [51]. Our analyses did not show the presence of inner membrane markers in OMVs composition including NADH-cytochrome C-oxidoreductase or succinate dehydrogenase.

In our study, outer membrane proteins with an OmpA motif were identified; these have been involved in immunostimulatory activities and induce leukocyte migration [54]. The presence of the family Ton B-dependent receptor proteins could be an alternative mechanism for *Brucella *survival in nutrient-limiting conditions such as found in macrophages [18, 55]. Additionally, Ton B-dependent receptors have been involved in siderophore internalization [18].

Omp16, and the Omp19 are lipoproteins that induce immunological protection very similarly to that elicited by the live vaccine *B. abortus* S19 with the induction of IFN-*γ* and CD4^+^ as well as CD8^+^ T-cells [29, 48, 56]. Also, the Omp16 shows significant similarity to the peptidoglycan-associated lipoprotein (PALs) of many Gram-negative bacteria [57].

Lipoproteins Omp10, Omp16 and Omp19 were previously identified in OMVs from *B. abortus* by Western blot [58].

OMVs from *B. melitensis* 16 M contain Cu-Zn SOD, Dps, and GroES that are part of the antioxidant defense system that protects bacteria from the toxic effects of reactive oxygen intermediates (ROIs) [59, 60]. The Dps protein has been reported in other pathogens (*Escherichia coli*, *Campylobacter jejuni,* and *Salmonella enterica)* to be responsible for resistance to oxidative stress and protecting the DNA against ROIs. The Dps protein has a ferritin-like domain (Table 1) and is thought to nullify the toxic combination of Fe (II) and peroxide [60, 61].

## 5. Conclusion

In summary, we identified 29 proteins in OMVs released by *B. melitensis *16 M, some of them are well-known *Brucella* immunogens such as SOD, GroES, Omp31, Omp25, Omp19, bp26, and Omp16. Additionally, we determined that rough OMVs both stimulate a stronger innate response, as well as protective immunity against *B. melitensis* 16 M challenge. Based on these data, the potential of using rough OMVs of *Brucella* as an acellular vaccine should be considered.

## Supplementary Material

Table S1. Results or the real time PCR values for each cytokine and each time point
Table S2. List of all proteins identified in OMVs
Table S3. In silico analysis and annotation of identified proteins based on hypothetical functions

## Figures and Tables

**Table 1:** *B. melitensis* 16 M OMVs proteins identified by 1D-SDS-PAGE coupled to LC-MS/MS.

Protein	*B. melitensis* denomination	Molecular weight (kDa)	Motif, subcellular localization	Closest ortholog, other than in Brucellae (% similarity)
Bacterial surface antigen	BMEI0830	85.90	Surface antigen, OM	Outer membrane protein *Bartonella henselae* str. Houston-1, (75.3%)
Iron-regulated outer membrane protein FRPB	BMEII0105	72.05	TonB-dependent receptor Plug Domain, OM	TonB-dependent receptor *Escherichia coli*, (41.3%)
Metal chelate outer membrane receptor	BMEI0657	64.80	TonB-dependent receptor Plug Domain, OM	Chain A, Outer Membrane Cobalamin Transporter (Btub) *Escherichia coli*, (46.2%)
Sugar-binding protein	BMEII0590	43.20	Bacterial sugar-binding extracellular protein, P	Probable sugar ABC transporter substrate binding protein *Rhizobium etli*, (75.7%)
Outer surface protein	BMEII0376	31.55	Surface antigen, OM	Probable heat-resistant agglutinin 1 protein *Rhizobium leguminosarum* bv. viciae, (54.9%)
D-Ribose-binding periplasmic protein precursor	BMEII0435	30.99	Periplasmic binding protein and sugar-binding domain of LacI family, P	Porin *Rhizobium leguminosarum* bv. trifolii, (59.2%)
Hypothetical protein BMEI0542	BMEI0542	30.04	Unknown, EC	Hypothetical protein *Rhizobium sp*., (48.3%)
25 kDa outer-membrane immunogenic protein precursor	BMEI1007	25.24	Porin type 2, OM	Hemin-binding C protein *Bartonella tricoborum*, (58.7%)
25 kDa outer-membrane immunogenic protein precursor	BMEI1249	23.18	Porin type 2, OM	Hemin-binding B protein *Bartonella henselae* str. Houston-1, (58.7%)
25 kDa outer-membrane immunogenic protein precursor	BMEI1829	24.58	Porin type 2, OM	Hemin-binding C protein *Bartonella tricoborum*, (45.4%)
25 kDa outer-membrane immunogenic protein precursor	BMEI1830	24.74	Porin type 2, OM	Outer membrane protein *Rhizobium etli*, (60.1%)
31 kDa outer-membrane immunogenic protein precursor	BMEII0844	23.27	OmpA-like domain profile, OM	Porin *Rhizobium leguminosarum* bv. trifolii, (52.5%)
BP26	BMEI0536	24.77	Protein of unknown function (DUF541), P	Unknown function protein DUF541 *Rhizobium leguminosarum* bv. trifolii, (64.9%)
Precursor YBIS protein	BMEI1369	23.51	Domain YkuD, C	Hypothetical protein *Rhizobium leguminosarum* bv. viciae, (67.2%)
OmpA family protein	BMEI0786	22.96	OmpA-like domain, OM	OmpA family protein *Rhizobium leguminosarum* bv. viciae, (81.4%)
Hypothetical lipoprotein	BMEI0785	21.91	Prokaryotic membrane lipoprotein lipid attachment profile, IM	Hypothetical protein *Rhizobium leguminosarum* bv. viciae, (74.7%)
Ribosome recycling factor	BMEI0826	20.66	Ribosome recycling factor (RRF-frr), C	Ribosome recycling factor *Bartonella henselae* str. Houston-1, (90.3%)
Hypothetical membrane-associated protein (BMEII0692)	BMEII0692	20.42	Invasion-associated locus B (IalB) protein, IM	Invasion-associated locus B family protein *Rhizobium leguminosarum* bv. trifolii, (62.4%)
22 kDa outer membrane protein precursor	BMEI0717	19.44	Unknown, OM	Outer membrane protein putative precursor *Rhizobium leguminosarum* bv. viciae, (48.6%)
DNA starvation/stationary phase protection protein Dps	BMEI1980	18.25	Dps protein family Ferritin-like domain, C	DNA starvation/stationary phase protection protein Dps *Escherichia coli*, (72.5%)
Peptidoglycan-associated lipoprotein	BMEI0340	18.23	OmpA family protein, OM	Outer membrane lipoprotein *Rhizobium etli*, (82.6%)
Invasion protein B	BMEI1584	18.03	Invasion-associated locus B (IalB) protein, P	Invasion-associated locus B protein *Bartonella quintana* str. Tolouse, (55.4%)
Outer membrane lipoprotein	BMEI0135	17.60	Bacterial outer membrane lipoprotein; Omp19, OM	Outer membrane lipoprotein *Bartonella henselae* str. Houston-1, (59.6%)
Chain A, Cu-Zn superoxide dismutase	BMEII0581	16.07	Cu-Zn superoxide dismutase, P	Cu-Zn superoxide dismutase *Escherichia coli*, (68.7%)
Thioredoxin C-1	BMEI2022	11.42	Thioredoxin active site, C	Putative thioredoxin *Rhizobium leguminosarum* bv. viciae, (88.8%)
Cochaperonin GroES	BMEII1047	10.39	10 kDa chaperonin protein Cnp10, C	Cochaperonin GroES *Bartonella tricoborum*, (91.8%)
DNA-binding protein HU	BMEI0877	9.07	Bacterial histone-like DNA binding protein signature, C	DNA-binding protein *Rhizobium sp*., (83.5%)
Hypothetical lipoprotein	Unknown	8.286	Prokaryotic membrane lipoprotein lipid attachment profile, OM	17 kDa surface antigen *Rhizobium leguminosarum* bv. trifolii, (61.2%)
Hypothetical protein BMEI0287	BMEI0287	8.596	Prokaryotic membrane lipoprotein lipid attachment profile, OM	Hypothetical protein *Rhizobium etli*, (56.8%)
