# Supplementary material for: Characterization of Outer Membrane Vesicles from Brucella melitensis and Protection Induced in Mice
Source: Clin Dev Immunol. 2011 Dec 29;2012:352493. doi: 10.1155/2012/352493 (PMC3254011; doi:10.1155/2012/352493)
Supplement: Supplementary file 1 — Table S1. Results or the real time PCR values for each cytokine and each time point Table S2. List of all proteins identified in OMVs Table S3. In silico analysis and annotation of identified proteins based on hypothetical functions [file 352493.f1.pdf]

Table S1.Cytokine expression of dendritic cells after stimulation with OMVs purified from *B. melitensis* 16M and *B. melitensis* VTRM1. The cytokine expression is reported as fold change in mRNA expression

OMVs from *B. melitensis* VTRM1		OMVs from *B. melitensis* 16M			
Time (h)	Cytokine fold change IFNγ	Time (h)	Cytokine foldchange IFNγ	P value	P value per group
1	11.27	1	0.4467	0.05	0.05
3	6.111	3	1.007	Ns	
6	0.7433	6	1.537	Ns	
12	7.453	12	5.18	Ns	
Time (h)	Cytokine fold change IL-2	Time (h)	Cytokine fold change IL-2	P value	P value per group
1	19.12	1	0.16	0.05	Ns
3	7.653	3	1.49	Ns	
6	1.243	6	4.52	Ns	
12	8.463	12	8.733	Ns	
Time (h)	Cytokine fold change IL-6	Time (h)	Cytokine fold change IL-6	P value	P value per group
1	61.37	1	0.3567	0.001	Ns
3	7.253	3	5.753	Ns	
6	4.633	6	12.54	Ns	
12	5.267	12	67.37	Ns	
Time (h)	Cytokine fold change IL-12	Time (h)	Cytokine fold change IL-12	P value	P value per group
1	53.83	1	0.2767	0.001	0.05
3	5.6	3	1.653	Ns	
6	1.277	6	6.087	Ns	
12	1.16	12	9.6	Ns	
Time (h)	Cytokine fold change IL-4	Time (h)	Cytokine fold change IL-4	P value	P value per group
1	1.572	1	0.1867	Ns	Ns
3	3.343	3	0.6933	Ns	
6	1.353	6	3.197	Ns	
12	0.1433	12	13.38	Ns	
Time (h)	Cytokine fold change IL-10	Time (h)	Cytokine fold change IL-10	P value	P value per group
1	2.36	1	1.007	Ns	Ns
3	9.457	3	4.62	Ns	
6	4.33	6	25.71	Ns	
12	3.193	12	41.54	Ns	

Time (h)	Cytokine fold change IL-17	Time (h)	Cytokine fold change IL-17	P value	P value per group
1	11.27	1	0.16	0.05	Ns
3	6.09	3	0.44	Ns	
6	0.07667	6	2.24	Ns	
12	7.453	12	14.54	Ns	
Time (h)	Cytokine fold change TNFα	Time (h)	Cytokine fold change TNFα	P value	P value per group
1	71.5	1	0.8109	0.05	0.001
3	100.7	3	2.778	0.05	
6	56	6	11.2	0.05	
12	59.33	12	19.17	Ns	
Time (h)	Cytokine fold change TGFβ	Time (h)	Cytokine fold change TGFβ	P value	P value per group
1	26.5	1	0.1754	0.01	Ns
3	4.145	3	0.04006	Ns	
6	3.295	6	4.862	Ns	
12	13.71	12	10.74	Ns	
Time (h)	Cytokine fold change IL-23	Time (h)	Cytokine fold change IL-23	P value	P value per group
1	18.96	1	0.0003104	0.05	Ns
3	0.1574	3	0.001534	Ns	
6	0.06513	6	0.1167	Ns	
12	1.736	12	0.2051	Ns	

Table S2. *B. melitensis* 16M OMVs proteins identified by 1D-SDS-PAGE coupled to LC-MS/MS

Gel section	Protein	Gene	Accession number (a.a)	Score (%)*	Coverage (%)**
1	Bacterial surface antigen	*omp1*	YP_221860	71	4
	Iron-Regulated outer membrane protein FRPB	*bhuA*	NP_541082	100	13
	Metal chelate outer membrane receptor	ND	NP_539574	90	3
2	Sugar binding protein	*sp39*	NP_541568	90	7
	Outer surface protein	ND	YP_223106	91	18
	D-Ribose binding periplasmic protein precursor	*rbsB*	NP_541413	99	50
	Hypothetical protein BMEI0542	ND	NP_539459	99	42
3	25 kDa outer-membrane immunogenic protein precursor	*omp2b*	NP_539924	99	28
	25 kDa outer-membrane immunogenic protein precursor	*omp25*	NP_540166	100	19
	25 kDa outer-membrane immunogenic protein precursor	*omp25c*	NP_540746	96	18
	25 kDa outer-membrane immunogenic protein precursor	*omp25d*	NP_540747	99	50
	31 kDa outer-membrane immunogenic protein precursor	*omp31*	NP_541822	99	32
	BP26	*bp26*	ABC17803	91	10
	Protein YBIS precursor	ND	AAL52550.	100	22
	OmpA family protein	*ompA*	NP_698208	99	17
4	Ribosome recycling factor	*frr*	NP_698164	100	38
	22 kDa outer-membrane immunogenic protein precursor	*omp22*	NP_539634	100	43
	DNA starvation/stationary phase protection protein Dps	*dps*	NP_699124	100	25
	Hypothetical lipoprotein	ND	NP_539702	90	14
5	Hypothetical Membrane Associated Protein (BMEII0692)	*ialB*	NP_541670	90	14
	Invasion protein B	*invB*	YP_221125	53	12
	Peptidoglycan-associated lipoprotein	*omp16*	NP_539257	99	40
	Chain A , -Cu-Zn superoxide dismutase	*sodC*	2AQM_A	100	48
	Co-chaperonin GroES	*groES*	NP_542025	99	42
	Thioredoxin C-1	*trxC*	NP_540939	98	16
6	Outer membrane lipoprotein	*omp19*	NP_539053	97	26
	DNA-binding protein HU	*hu*	YP_221813	95	20
	Hypothetical lipoprotein	ND	YP_221486	100	43
	Hypothetical protein BMEI0287	ND	NP_539204	100	50

ND. None designated
* The percentage of detection is the value assigned on the identity of a protein. **The percentage of coverage corresponds to the length of the sequence covering by the peptides obtained in the LC-MS/MS for this sequence.

Table S3. *In silico* analyses of proteins and annotation of identified vesicular proteins based on hypothetical function by software database (ProLinks and Gene Ontology).

**Identified protein**	**Antigenic region**	**I. P.**	***Signal peptide**	**Related to (with ProLinks)**	**GO terms (Process or function)**
Bacterial surface antigen	[+7.627]:SGRRLRDVI; [+5.862]:ETLRRFYYN; [+4.107]:LSAGFDVFR	5.37	Yes	Membrane proteins	Membrane, transmembrane, outer membrane
Iron-Regulated outer membrane protein FRPB	[+6.969]:PGKTFGARI; [+3.994]:AGASRTWLG [+4.461]:APGRTFTFQ	5.54	Yes	Membrane proteins	Membrane, transmembrane, outer membrane
Metal chelate outer membrane receptor	[+6.223]: RTASATSGS; [+6.164]: LQGSFNFAL [+5.277]: QYDATAPAF;[+8.431]: TIDYDETGR	5.00	No	Membrane proteins	Receptor, transporter, membrane
Sugar binding protein	[+5.349]:IESPAFQSA; [+11.014]: AIYDVVTRQ	4.97	Yes	Sugar transport	Sugar transport, periplasmic
Outer surface protein	[+5.263]:GAGIGAAYV; [+4.550]:YGYDEQDRQ [+4.643]:VINQYGQGA	5.28	Yes		Membrane, transmembrane, outer membrane
D-Ribose binding periplasmic protein precursor	[+10.821]:AANIPVFLI; [+4.572]:ANIPVFLIN [+2.382]:ANWDRTQGH	5.60	No	Sugar transport	D-ribose transport, outer membrane-bounded periplasmic space, sugar binding
Hypothetical protein BMEI0542	[+3.159]:SKVEWTDPF; [+3.446]:DINDRWNIF [+4.666]:DIGGFGAGT	4.83	Yes		
25 kDa outer-membrane immunogenic protein precursor	[+5.837]:GVDSKLRWS;[+3.644]:DFSVVDGDL	4.72	Yes	Membrane proteins	Membrane, transmembrane, outer membrane
25 kDa outer-membrane immunogenic protein precursor	[+3.213]:WAGGYTGLY;[+12.005]:NYDLAGTTV	8.58	Yes		Membrane, transmembrane, outer membrane
25 kDa outer-membrane immunogenic protein precursor	[+2.813]:ATGANAADA;[+2.005]:APVVVAPTF	4.78	Yes	Membrane proteins	Membrane, transmembrane, outer membrane
25 kDa outer-membrane immunogenic protein precursor	[+4.891]:EPAPIAIAP; [+4.062]:NNLKKSRDF	9.28	Yes	Membrane proteins	Membrane, transmembrane, outer membrane
31 kDa outer-membrane immunogenic protein precursor	[+5.082]:KHPFSSFDK;[+4.300]: RLGYTATER	5.21	Yes	Membrane proteins	Membrane, transmembrane, outer membrane
BP26	[+1.069]: TMLAAAPDN**	7.93	Yes		Periplasmic space
Precursor YBIS protein	[+2.858]:KRQWPRWP;[+8.867]:ALYIFKDGK	8.49	No		
OmpA family protein	[+4.455]:GAGIGALGG;[+2.212]:DTDQDQVKS	9.55	Yes		Outer membrane component, transmembrane
Hypothetical lipoprotein	[+6.979]: RGGWFNNQ; [+7.882]: DAIHFIGWY [+5.847]ADAIHFIGW	10.48	Yes		Lytic endotransglycosylase activity
Ribosome recycling factor	[+4.144]:RIAARHVRR	5.98	No	Translation	Translation, translation termination
Hypothetical membrane-associated protein(BMEII0692)	[+2.166]: MLASATMPA;[+2.363]: PNPIPVALT	9.00	No		
22 kDa outer membrane protein precursor	[+7.382]: QVNGIEQRN	8.59	No	Membrane proteins	Membrane, transmembrane, outer membrane
DNA starvation/stationary phase protection protein Dps	[+7.137]: ITKQAHWNL;[+8.278]: RLKPYPTDI [+4.828]: LKPYPTDIY	5.25	No	Aminoacid transport	Metal ion binding, redox activity, ferric iron binding
Peptidoglycan associated lipoprotein	[+8.084]: ASKKNLPNN;[+3.394]: RTISYGNER	9.92	Yes	Toluene transport	Membrane, transmembrane, outer membrane
Invasion protein B	[+4.700]:KIDDTAGPN;[+2.813]:QETYQDWTV	8.98	Yes		Membrane
Outer membrane lipoprotein	[+5.631]: ASAPDLTPG;[+4.541]: ISKASLLSL [+4.923] : LLSLAAAGI	8.91	Yes		Membrane, transmembrane, outer membrane
Chain A, Cu-Zn superoxide dismutase	[+1.343]: TGPGKEVGT**	6.11	No		Ion binding, redox activity
Thioredoxin C-1	[+10.205]:WAEWCGPK; [+3.554]: MFKDGELA	4.53	No	A transketolase	Cellular protein metabolic process, generation of precursor metabolites and energy, nucleotide metabolic process
Co-chaperonin GroES	[+4.231]:VRRVESEAK	5.41	No		Protein folding, chaperone binding, chaperone regulator activity
DNA binding protein HU	[+1.540]: AEKGGLTKA**	9.77	No	The primosome	DNA binding
Hypothetical lipoprotein	[+2.847]: AGGAIIGGI	10.50	Yes		
Hypothetical protein BMEI0287	[+2.729]: RTAGYGVGG	9.02	Yes		

I. P. Isoelectric point.

*Signal P: used for the identification of the signal peptide into the protein sequence.

** Unique antigenic region into the sequence.
